# Supplementary material for: The Greatwall–Endosulfine Switch Accelerates Autophagic Flux during the Cell Divisions Leading to G1 Arrest and Entry into Quiescence in Fission Yeast
Source: Int J Mol Sci. 2022 Dec 21;24(1):148. doi: 10.3390/ijms24010148 (PMC9820488; doi:10.3390/ijms24010148)
Supplement: Supplementary file 1 [file ijms-24-00148-s001.zip › ijms-2045788-supplementary.pdf]

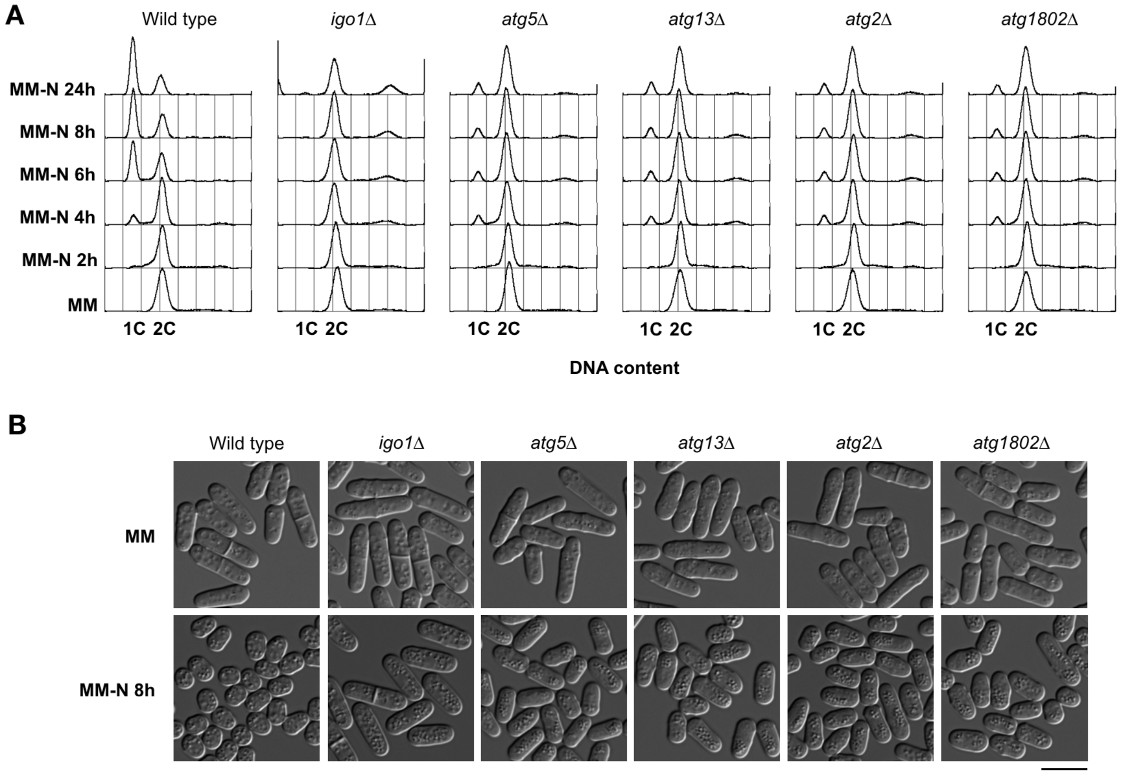

Supplementary Figure S 1. Autophagy-related genes are required for the last two cell divisions preceding G1 arrest after nitrogen starvation. A) Flow cytometry analysis of wild type, *igo1Δ*, *atg5Δ*, *atg13Δ*, *atg2Δ* and *atg1802Δ* grown at 25ºC in minimal medium (MM) and after 2, 4, 6, 8 and 24 hours in nitrogen-free minimal medium (MM-N). **B)** Representative Nomarski images of the cells grown at 25ºC in MM and after 8 hours in MM-N. Scale bar 10 µM.

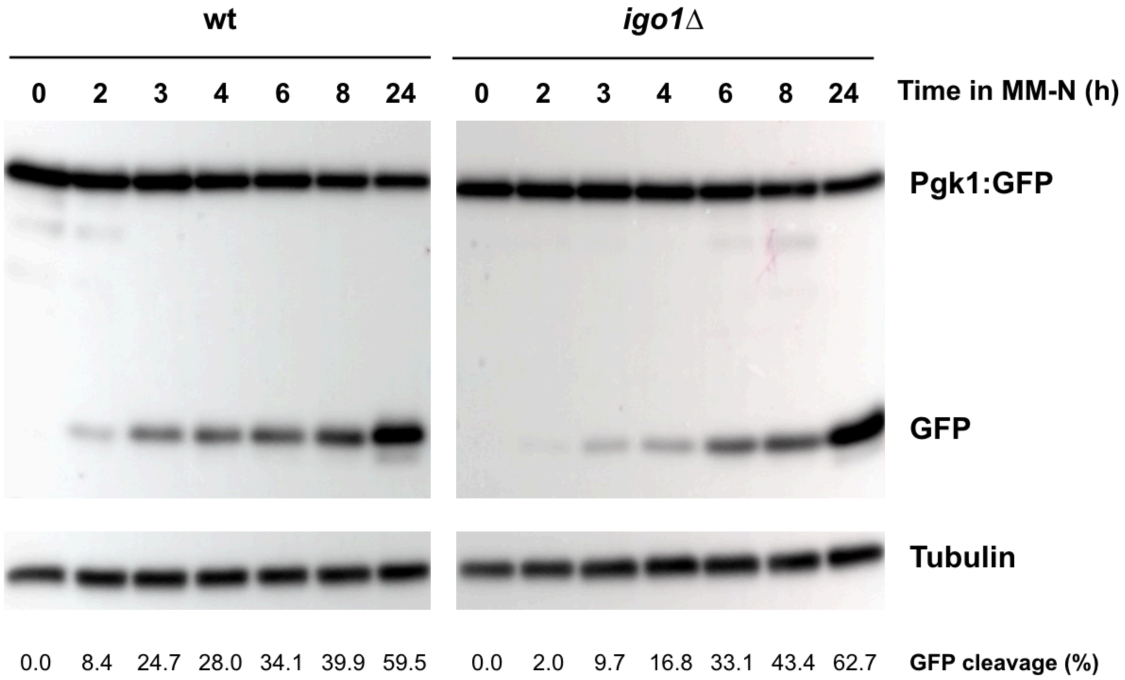

Supplementary Figure S 2. Endosulfine accelerates autophagic flux. Wild type and *igo1Δ* cells expressing *pgk1:GFP* were grown in minimal medium at 25ºC until mid-exponential phase and then transferred to nitrogen-deprived minimal medium (MM-N) for 24 hours at 25ºC. Samples were taken at 0, 2, 4, 6, 8 and 24 hours in MM-N. Pgk1:GFP and free GFP levels were determined using anti-GFP antibodies (Chromotek). Tubulin was used as a loading control. The percentage of GFP cleavage is indicated under the gels.

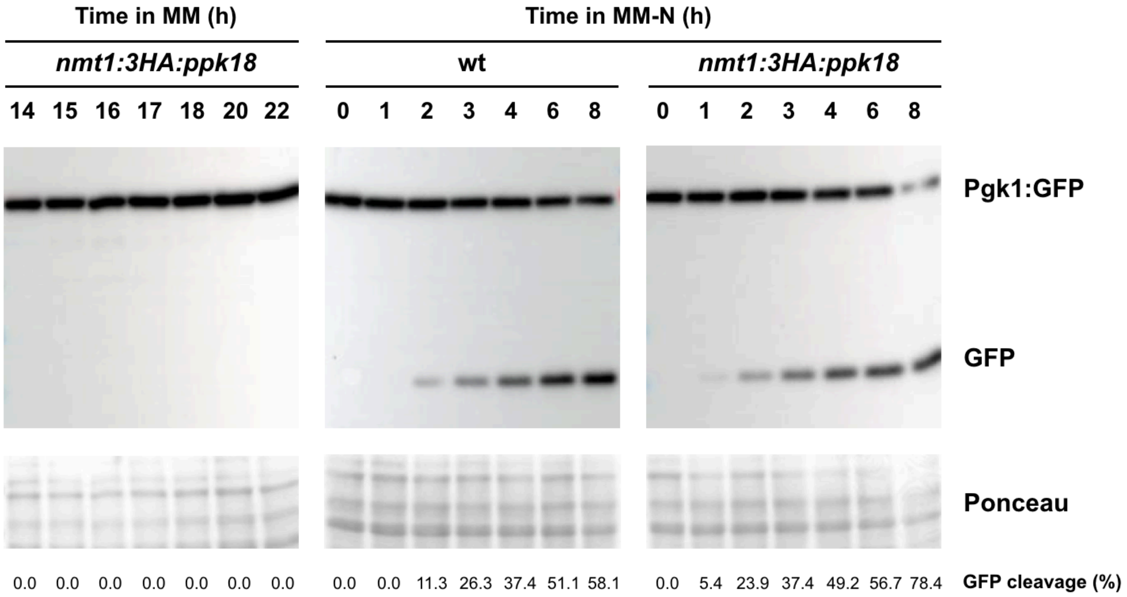

Supplementary Figure S3. Overexpression of Greatwall accelerates autophagic flux. Wild type and cells expressing *ppk18⁺* under the *nmt1* promoter *(nmt1:3xHA:ppk18⁺*) expressing Pgk1:GFP grown in minimal medium with thiamine at 25ºC until mid-exponential phase were transferred to minimal medium without thiamine to induce the expression of *ppk18⁺*. After 14 hours at 25ºC, part of the cultures (panels middle and right) was transferred to nitrogen-free minimal medium (MM-N) for 8 hours at 25ºC. Samples were taken at 14, 15, 16, 17, 18, 20 and 22 hours in MM and at 0, 1, 2, 3, 4, 6 and 8 hours in MM-N. Pgk1:GFP and free GFP levels were determined using anti-GFP antibodies (Chromotek). Ponceau staining was used as a loading control. The percentage of GFP cleavage is indicated under the gels.

Supplementary Table S1. Autophagy-related coding genes upregulated in *nmt1:ppk18*$^{+}$ ON compared to the wild type. In red genes upregulated more than two-fold.

systematic	baseMean	log2FoldChange	lfcSE	stat	pvalue	padj	synonyms
SPAC19G12.10c	63252.27	1.38	0.07	19.05	6.50E-81	1.82E-79	cpy1
SPBC1711.11	236.88	1.32	0.20	6.71	1.94E-11	4.53E-11	atg2402
SPAC14C4.01c	1197.06	1.27	0.11	11.91	1.00E-32	9.35E-32	atg43
SPCC63.08c	2062.86	1.20	0.10	12.18	4.15E-34	5.81E-33	atg1
SPAC4F10.07c	1458.14	1.12	0.10	11.37	5.66E-30	3.96E-29	atg13
SPAC589.07c	384.72	1.08	0.16	6.82	9.22E-12	2.35E-11	atg1801
SPAC19B12.08	443.94	1.08	0.15	7.29	3.13E-13	1.10E-12	atg4
SPCC16A11.08	463.94	1.05	0.15	7.19	6.71E-13	2.09E-12	atg20
SPBC6B1.05c	752.16	1.02	0.12	8.50	1.93E-17	9.01E-17	atg7
SPAC10F6.11c	1121.62	0.89	0.11	8.20	2.48E-16	9.91E-16	atg17
SPBP35G2.11c	1981.79	0.80	0.09	8.59	8.34E-18	4.67E-17	nbr1
SPAC823.16c	478.93	0.76	0.14	5.28	1.27E-07	2.37E-07	atg1802
SPAC23C4.16c	1095.84	0.75	0.10	7.15	8.71E-13	2.44E-12	atg15
SPAC25A8.02	746.90	0.70	0.12	5.70	1.18E-08	2.54E-08	atg14
SPBP8B7.24c	697.72	0.70	0.13	5.56	2.64E-08	5.27E-08	atg8
SPAC20G8.10c	298.39	0.66	0.17	3.80	0.0001	0.0002	atg6
SPAC2G11.13	789.80	0.64	0.13	4.96	6.90E-07	1.21E-06	atg22
SPBC15D4.07c	888.23	0.42	0.11	3.73	0.0002	0.0003	atg9
SPBC31E1.01c	2168.06	0.30	0.09	3.42	0.0006	0.0009	atg2

Supplementary Table S2. Autophagy-related coding genes downregulated in *igo1Δ* compared to the wild type in MM-N 1h. In red genes downregulated more than two-fold.

systematic	baseMean	log2FoldChange	lfcSE	stat	pvalue	padj	synonyms
SPBC660.08	1642.86	0.20	0.07	2.90	0.0038	0.0046	atg38
SPBC31E1.01c	2685.31	-0.14	0.05	-2.63	0.0086	0.0097	atg2
SPAC19G12.10c	95221.26	-0.20	0.07	-2.71	0.0067	0.0078	cpy1
SPAC7D4.04	2772.77	-0.26	0.04	-6.68	2.39E-11	3.94E-11	atg11
SPBC4B4.10c	622.41	-0.28	0.06	-4.61	4.06E-06	5.68E-06	atg5
SPAC6F6.12	2299.97	-0.34	0.04	-7.63	2.39E-14	4.78E-14	atg24
SPAC25A8.02	1088.91	-0.39	0.05	-7.15	8.38E-13	1.57E-12	atg14
SPAC14C4.01c	2507.19	-0.40	0.04	-9.40	5.43E-21	1.38E-20	atg43
SPBC405.05	290.83	-0.41	0.10	-4.05	5.05E-05	6.74E-05	atg16
SPAC823.16c	790.15	-0.45	0.06	-7.84	4.45E-15	9.58E-15	atg1802
SPBP35G2.11c	2556.03	-0.50	0.14	-3.68	0.0002	0.0003	nbr1
SPAC4F10.07c	2233.62	-0.51	0.07	-7.02	2.19E-12	3.83E-12	atg13
SPAC458.06	342.94	-0.51	0.08	-6.02	1.75E-09	2.72E-09	atg1803
SPBC15D4.07c	1238.49	-0.60	0.06	-10.82	2.64E-27	7.38E-27	atg9
SPBC6B1.05c	1318.21	-0.62	0.06	-11.11	1.11E-28	3.46E-28	atg7
SPAC10F6.11c	1464.91	-0.65	0.05	-13.99	1.70E-44	9.53E-44	atg17
SPAC19B12.08	865.42	-0.70	0.08	-8.29	1.14E-16	2.65E-16	atg4
SPAC20G8.10c	443.82	-0.78	0.07	-11.11	1.11E-28	3.46E-28	atg6
SPCC63.08c	3165.31	-0.81	0.13	-5.99	2.07E-09	3.06E-09	atg1
SPAC23C4.16c	1709.38	-0.89	0.06	-14.16	1.63E-45	1.14E-44	atg15
SPAC589.07c	462.59	-0.89	0.07	-12.42	1.96E-35	7.85E-35	atg1801
SPAC2G11.13	1717.04	-0.97	0.05	-19.05	6.72E-81	1.88E-79	atg22
SPBC1711.11	580.25	-1.00	0.08	-13.13	2.18E-39	1.02E-38	atg2402
SPBP8B7.24c	1194.86	-1.03	0.06	-17.69	5.47E-70	7.66E-69	atg8
SPCC16A11.08	716.47	-1.08	0.07	-15.00	7.40E-51	6.90E-50	atg20

Supplementary Table S3. Autophagy-related coding genes downregulated in *ppk18Δ cek1Δ* compared to the wild type in MM-N 1h.

systematic	baseMean	log2FoldChange	lfcSE	stat	pvalue	padj	synonyms
SPBC660.08	1642.8595	0.2411	0.0705	3.4204	0.0006	0.0008	atg38
SPBC31E1.01c	2685.3116	-0.1598	0.0526	-3.0396	0.0024	0.0028	atg2
SPAC7D4.04	2772.7651	-0.2813	0.0385	-7.3053	2.77E-13	5.53E-13	atg11
SPAC6F6.12	2299.9662	-0.3178	0.0450	-7.0631	1.63E-12	3.04E-12	atg24
SPAC14C4.01c	2507.1859	-0.3349	0.0430	-7.7823	7.12E-15	1.53E-14	atg43
SPBC4B4.10c	622.4129	-0.3584	0.0611	-5.8698	4.36E-09	6.43E-09	atg5
SPAC823.16c	790.1464	-0.3635	0.0570	-6.3720	1.87E-10	3.07E-10	atg1802
SPBC405.05	290.8314	-0.3794	0.1012	-3.7487	0.0002	0.0002	atg16
SPAC25A8.02	1088.9127	-0.4269	0.0542	-7.8746	3.42E-15	7.98E-15	atg14
SPAC4F10.07c	2233.6229	-0.4960	0.0721	-6.8778	6.08E-12	1.06E-11	atg13
SPAC458.06	342.9437	-0.5006	0.0849	-5.8966	3.71E-09	5.77E-09	atg1803
SPBP35G2.11c	2556.0320	-0.5170	0.1368	-3.7794	0.0002	0.0002	nbr1
SPBC15D4.07c	1238.4939	-0.5371	0.0556	-9.6630	4.33E-22	1.21E-21	atg9
SPBC6B1.05c	1318.2146	-0.6160	0.0564	-10.9256	8.69E-28	2.70E-27	atg7
SPAC10F6.11c	1464.9122	-0.6171	0.0467	-13.2141	7.27E-40	5.09E-39	atg17
SPAC23C4.16c	1709.3795	-0.7155	0.0626	-11.4374	2.72E-30	9.52E-30	atg15
SPAC19B12.08	865.4175	-0.7301	0.0849	-8.6015	7.87E-18	2.00E-17	atg4
SPCC63.08c	3165.3128	-0.7817	0.1346	-5.8070	6.36E-09	8.90E-09	atg1
SPAC20G8.10c	443.8231	-0.8146	0.0711	-11.4657	1.96E-30	8.39E-30	atg6
SPAC589.07c	462.5883	-0.8433	0.0717	-11.7552	6.64E-32	3.72E-31	atg1801
SPBC1711.11	580.2515	-0.8702	0.0759	-11.4599	2.10E-30	8.39E-30	atg2402
SPAC2G11.13	1717.0446	-0.9295	0.0511	-18.1928	5.89E-74	1.65E-72	atg22
SPCC16A11.08	716.4699	-0.9931	0.0722	-13.7621	4.31E-43	4.02E-42	atg20
SPBP8B7.24c	1194.8589	-0.9976	0.0583	-17.1238	9.86E-66	1.38E-64	atg8

Supplementary Table S4. Strains used in this work.

Strain	*Genotype*	Source
S2337	h^+ *ppa2::natMX6*	S. Moreno
S2338	h^+ *igo1::kanMX6 ppa2::natMX6*	S. Moreno
S2341	h^- *ppk18::kanMX6*	S. Moreno
S2392	h^- *kanMX6:P41nmt1:GFP:ppk18*$^+$	S. Moreno
S2431	h^+ *kanMX6:P41nmt1:GST:pab1*$^+$	S. Moreno
S2432	h^+ *igo1::kanMX6 kanMX6:P41nmt1:GST:pab1*$^+$	S. Moreno
S2641	h^- *CFP:atg8:leu1*$^+$ *leu1-32*	Li-Lin Du DY3510
S2726	h^- *972*	S. Moreno
S2727	h^- *igo1::kanMX6*	S. Moreno
S2876	h^- *ppk31::hphMX6*	S. Moreno
S2878	h^- *cek1::natMX6*	S. Moreno
S2881	h^- *ppk18::kanMX6 ppk31::hphMX6*	S. Moreno
S2883	h^- *ppk18::kanMX6 cek1::natMX6*	S. Moreno
S2884	h^- *ppk31::hphMX6 cek1::natMX6*	S. Moreno
S2885	h^- *ppk18::kanMX6 ppk31::hphMX6 cek1::natMX6*	S. Moreno
S2980	h^- *atg2::kanMX6*	S. Moreno
S2981	h^- *atg13::kanMX6*	S. Moreno
S2982	h^- *atg5::kanMX6*	S. Moreno
S2983	h^- *atg1802::kanMX6*	S. Moreno
S2984	h^- *pgk1:L:GFP:kanMX6*	This work
S2985	h^- *pgk1:L:GFP:hphMX6*	This work
S2986	h^- *pgk1:L:GFP:kanMX6 igo1::natMX6*	This work
S2987	h^- *pgk1:L:GFP:hphMX6 kanMx6:P3nmt1:3HA:ppk18*$^+$	This work
S2988	h^- *CFP:atg8:leu1*$^+$ *kanMX6:P3nmt1:3HA:ppk18*$^+$ *leu1-32*	This work
S2999	h^- *CFP:atg8:leu1*$^+$ *igo1::kanMX6 leu1-32*	This work
S3000	h^- *CFP:atg8:leu1*$^+$ *ppa2::kanMX6 leu1-32*	This work
S3001	h^+ *CFP:atg8:leu1*$^+$ *igo1::natMX6 ppa2::kanMX6 leu1-32*	This work
S3002	h^- *CFP:atg8:leu1*$^+$ *pab1::kanMX6 leu1-32*	This work
S3003	h^- *CFP:atg8:leu1*$^+$ *kanMX6:P41nmt1:GST:pab1*$^+$ *leu1-32*	This work
S3004	h^- *CFP:atg8:leu1*$^+$ *ppk18::KanMX4 leu1-32*	This work
S3005	h^- *CFP:atg8:leu1*$^+$ *cek1::natMX6 leu1-32*	This work
S3006	h^- *CFP:atg8:leu1*$^+$ *ppk18::KanMX6 cek1::natMX6 leu1-32*	This work

S3007	*h⁻ CFP:atg8:leu1⁺ sck1::kanMX4 leu1-32*	This work
S3008	*h⁻ CFP:atg8:leu1⁺ sck2::natMX6 leu1-32*	This work
S3009	*h⁻ CFP:atg8:leu1⁺ psk1::kanMX4 leu1-32*	This work
S3012	*h^{90} CFP:atg8:leu1⁺ atg13::kanMX6 leu1-32*	This work
S3013	*h⁻ kanMX6:P3nmt1:GFP:ppk18⁺*	S. Moreno
S3021	*h⁻ CFP:atg8:leu1⁺ ppk31::hphMX6 leu1-32*	This work
S3022	*h⁻ CFP:atg8:leu1⁺ ppk18::kanMX6 ppk31::hphMX6 leu1-32*	This work
S3023	*h⁻ CFP:atg8:leu1⁺ cek1::natMX6 ppk31::hphMX6 leu1-32*	This work
